# Pleural Fluid Adenosine Deaminase (Pfada) in the Diagnosis of Tuberculous Effusions in a Low Incidence Population

**DOI:** 10.1371/journal.pone.0113047

**Published:** 2015-02-03

**Authors:** David T. Arnold, Rahul Bhatnagar, Lynette D. Fairbanks, Natalie Zahan-Evans, Amelia O. Clive, Anna J. Morley, Andrew R. L. Medford, Nicholas A. Maskell

**Affiliations:** 1 Academic Respiratory Unit, School of Clinical Sciences, University of Bristol, Bristol, United Kingdom; 2 North Bristol Lung Centre, Southmead Hospital, Bristol, United Kingdom; 3 Purine Research Laboratory, St Thomas’ Hospital, London, United Kingdom; Public Health Agency of Barcelona, SPAIN

## Abstract

**Introduction:**

Previous studies have assessed the diagnostic ability of pleural fluid adenosine deaminase (pfADA) in detecting tuberculous pleural effusions, with good specificity and sensitivity reported. However, in North Western Europe pfADA is not routinely used in the investigation of a patient with an undiagnosed pleural effusion, mainly due to a lack of evidence as to its utility in populations with low mycobacterium tuberculosis (mTB) incidence.

**Methods:**

Patients presenting with an undiagnosed pleural effusion to a tertiary pleural centre in South-West England over a 3 year period, were prospectively recruited to a pleural biomarker study. Pleural fluid from consecutive patients with robust 12-month follow up data and confirmed diagnosis were sent for pfADA analysis.

**Results:**

Of 338 patients enrolled, 7 had confirmed tuberculous pleural effusion (2%). All mTB effusions were lymphocyte predominant with a median pfADA of 72.0 IU/L (range- 26.7 to 91.5) compared to a population median of 12.0 IU/L (range- 0.3 to 568.4). The optimal pfADA cut off was 35 IU/L, which had a negative predictive value (NPV) of 99.7% (95% CI; 98.2-99.9%) for the exclusion of mTB, and sensitivity of 85.7% (95% CI; 42.2-97.6%) with an area under the curve of 0.88 (95% CI; 0.732–1.000).

**Discussion:**

This is the first study examining the diagnostic utility of pfADA in a low mTB incidence area. The chance of an effusion with a pfADA under 35 IU/L being of tuberculous aetiology was negligible. A pfADA of over 35 IU/L in lymphocyte-predominant pleural fluid gives a strong suspicion of mTB.

## Introduction

Pleural fluid adenosine deaminase (pfADA) measurement is commonly used in countries with a moderate to high incidence of mycobacterium tuberculosis (mTB). In these areas it is often used routinely in the investigation of undiagnosed pleural effusions, or to supplement standard pleural fluid analysis where a tuberculous effusion is suspected. ADA is a purine catabolic enzyme that catalyses the conversion of adenosine to inosine and is particularly abundant in lymphoid tissue[1]. In comparison to the other investigations for mTB, pfADA can be quickly analysed and is relatively non-invasive to obtain with thoracocentesis. Direct visualisation of acid fast bacilli in pleural fluid has low yield rates of between 5–10%[2, 3] and culture results from pleural fluid and sputum only have a sensitivity of 50% and 30% respectively, as well as taking up to 8 weeks to perform[4, 5]. In countries where the BCG was used nationally the Mantoux test has limited utility. The gold standard for diagnosis of pleural mTB is thoracoscopic pleural biopsy, with pick-up rates approaching 100%[6], but in some patients this invasive test may be inappropriate.

In the UK, and other North-Western European countries, pfADA has limited use outside of specialist centres, in part due to a lack of evidence where mTB incidence is low[7]. We report a prospective study examining the utility of pfADA in an area with an mTB incidence of 7.8 per 100,000[8].

## Methods

### Patients

Consecutive patients referred to a tertiary pleural centre with an undiagnosed pleural effusion had blood, pleural fluid, and clinical and demographic information collected prospectively. The study received ethical approval from the South West regional ethics committee (REC number 08/H0102/11) and all patients provided written informed consent. In addition to routine baseline pleural investigations pleural fluid was centrifuged, and the supernatant stored at -70°c. All patients were assigned a final diagnosis at either death or 12 months (whichever came sooner). The final diagnosis was agreed by two independent respiratory consultant physicians based on all the available clinical information. Any areas of contention were re-examined to reach consensus. Notably, pfADA results were not available at the time of diagnosis.

### Diagnostic criteria

Pre-defined diagnostic criteria were used to reach a 12 month diagnosis. Tuberculous pleural effusion was diagnosed in the presence of any of the following: positive fluid or tissue culture for mycobacterium tuberculosis (mTB); acid fast bacilli confirmed in sputum, pleural fluid or pleural tissues; or in patients with a strong clinical and radiological suspicion of TB pleuritis who had a positive quantiferon and showed resolution of the presenting pleural effusion after 6 months anti-TB therapy. See S1 Appendix for full details and other diagnostic criteria.

### Pleural fluid analysis

Routine pleural fluid analysis was completed at the time of study enrolment and included protein, glucose, LDH, pH, microscopy, culture, cytology and flow cytometry with cytogenetics. Light’s criteria were used to distinguish exudative from transudative effusions[9]. Predominant pleural cell types were defined based on British Thoracic Society guidelines[10]. Specifically a lymphocyte-predominant effusion was defined as the presence of over 50% lymphocytes in the absence of any malignant cells or ≥10% eosinophils, in which case the effusion was deemed malignant or eosinophilic respectively.

pfADA was measured ‘en bloc’ post patient recruitment after a median of 41 months (range 25–63 months) of storage at -70°c, by a single investigator blinded to patient diagnosis. A photodiode array ultraviolet detector was used with a non-Giusti method. Before analysis (to reduce the risk of infection) all samples were thawed then filtered using individual Sterifix 0.2um luer lock filters in a category 3 laboratory and were refrozen before transit. See S2 Appendix for full details on pfADA analysis.

### Statistical analysis

Statistical analysis was performed using SPSS 21.0 statistical software (Chicago, IL, USA). pfADA was non-parametric so was described using medians and ranges. Demographic and biochemical factors affecting pfADA were assessed using multiple linear regression analysis with backwards selection. Variables with a p value >0.1 were removed from the model. The accuracy of pfADA as a diagnostic test was assessed using standard sensitivity, specificity, positive predictive values (PPV) and negative predictive values (NPV) with 95% confidence intervals.

## Results

### Patient demographics and aetiology of pleural effusions

338 consecutive patients with stored pleural fluid from undiagnosed pleural effusions were entered into a pleural biomarkers study between December 2008 and February 2012 and had their pleural fluid sent for ADA analysis. The cohort had a mean age of 70.6 (range 18–96) and was 65% male (218 male vs 120 female).

Table 1 shows the breakdown of all effusions, with malignant (51%) and parapneumonic (21%) aetiologies forming the majority. 79% (267/338) of all cause effusions were exudates as defined by Light’s criteria. Of 71 parapneumonic effusions, 12 were diagnosed as bacterial empyema and 39 were diagnosed as complicated parapneumonic effusions (CPPE). Reasons for CPPE diagnosis included low pleural pH or loculations on USS (n = 28), positive gram stain or culture of pleural fluid (n = 3), culture or histological findings of pleural biopsy (n = 4), and CT evidence of pleural infection with radiological evidence of resolution following treatment (n = 4).

**Table 1:** Aetiology of pleural effusion across the cohort (n-338).

Causes	N (%)
Malignancy	172 (51%)
Lung	56
MPM	42
Breast	24
Ovarian	14
Gastrointestinal	7
Lymphoma	8
Head and neck	5
Other	16
Transudates	44 (13%)
CCF	33
Hepatic	7
Renal	4
Parapneumonic	71 (21%)
Simple	20
Complicated	39
Empyema	12
Mycobacterium Tuberculosis	7 (2%)
Other exudates	39 (12%)
BAPE	15
Inflammatory pleuritis	11
Trauma	4
Pulmonary embolus	3
Other	6
Idiopathic	5 (1%)

BAPE- Benign Asbestos Related Pleural Effusion, CCF- Congestive Cardiac Failure, MPM- Malignant Pleural Mesothelioma.

Table 2 shows the details of the 7 patients diagnosed with a tuberculous effusion (2%) including basis for diagnosis and baseline demographic, biochemical and sensitivity data. Five had a culture positive diagnosis of mTB, 1 had granulomatous tissue seen at biopsy and a positive quantiferon, and 1 had a clinical and radiological suspicion of mTB with a positive quantiferon. All had resolution of their pleural effusion after 6 months of anti-TB therapy. No patients were found to have atypical mycobacteria or antibiotic resistance on culture. There were no cases of concurrent HIV infection, although two patients were receiving immunosuppressive therapy for autoimmune disease. Of the 7 patients with mTB all had lymphocyte predominant exudative pleural effusions.

**Table 2:** Details of patients diagnosed with tuberculous pleural effusion.

Age/Sex	Smoking status	pfADA (IU/L)	Predominant cell (% on differential)	Diagnostic evidence	Sensitivities to anti-TB antibiotics	Resolution with anti-TB therapy?
37 M	Never	89.0	Lymphocytic (85%)	Pleural biopsy, sputum and pleural fluid grew mTB	Fully	Yes
70 F	Never	37.9	Lymphocytic (60%)	Pleural biopsy, sputum and pleural fluid grew mTB	Fully	Yes
45 M	Never	72.0	Lymphocytic (100%)	Biopsy demonstrated granulomatous tissue. No growth on culture. Positive quantiferon	n/a	Yes
40 M	Never	73.3	Lymphocytic (70%)	Pleural biopsy grew mTB	Fully	Yes
81 M	Ex	91.5	Lymphocytic (95%)	Pleural fluid grew mTB	Fully	Yes
41 M	Current	26.7	Lymphocytic (75%)	BAL grew mTB	Fully	Yes
38 M	Never	35.0	Lymphocytic (80%)	Clinical and radiological suspicion of mTB but no growth on culture. Positive quantiferon.	n/a	Yes

BAL- bronchoalveolar lavage, mTB- mycobacterium tuberculosis.

### pfADA in all effusion types

**Fig 1:**
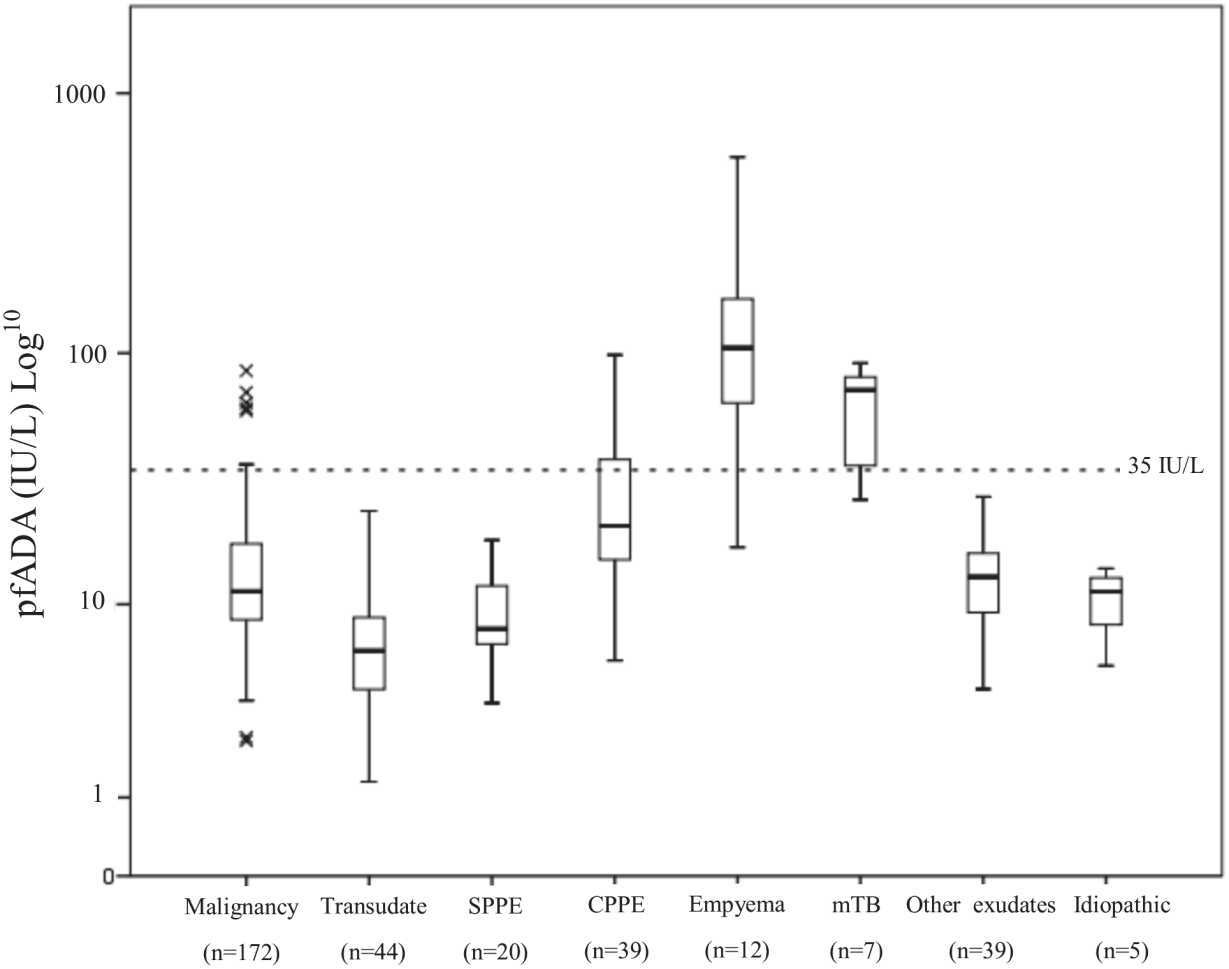
Boxplot of pfADA levels by diagnostic category for all effusions (n = 338). Abbreviations; SPPE—Simple parapneumonic effusion, CPPE—Complicated parapneumonic effusion, mTB—mycobacterium tuberculosis.

The median pfADA level was 12.0 IU/L (range 1.3 to 568.4). Fig. 1 shows the distribution of pfADA by aetiology for all effusions. Median levels of pfADA were highest in empyema (104.9 IU/L, range 17.2–568.4) followed by mTB (median 72.0 IU/L, range 26.7 to 91.5). Multiple linear regression was used to assess the effect of demographic, pleural and serum factors on pfADA levels. After backward elimination, patient age (R = -0.161, P = 0.049), pleural protein (R = 0.249, P = 0.002) and pleural LDH (R = 0.009, P<0.001) were independently linked to pfADA level. Notably there was no correlation between the storage time of pleural fluid and pfADA.

**Fig 2:**
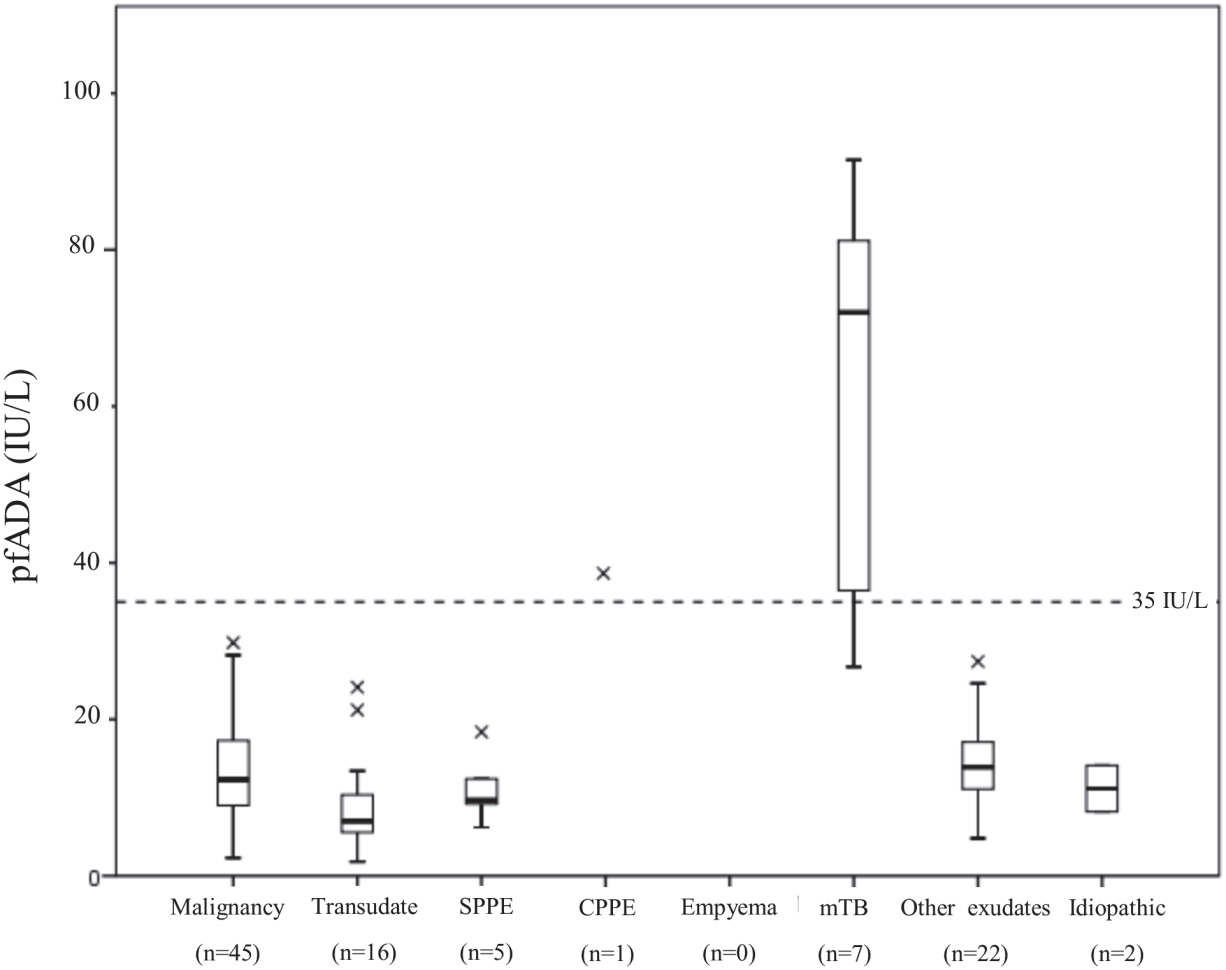
Boxplot of pfADA levels by diagnostic category for lymphocyte predominant effusions (n = 98). Abbreviations; SPPE—Simple parapneumonic effusion, CPPE—Complicated parapneumonic effusion, mTB—mycobacterium tuberculosis.

Based on previous literature[11] and confirmatory results from our receiver operating curve (ROC) a cut off of 35 IU/L (greater than or equal to) was chosen to differentiate ‘low’ from ‘high’ pfADA. Using this cut off there were 36 patients who fell into the high pfADA group, all of whom had exudative effusions (see Table 3). Of these, 7 were lymphocyte predominant with 6 tuberculous effusions and 1 CPPE (see Fig. 2). Two cases of non small cell lung cancer (NSCLC) and 4 cases of malignant pleural mesothelioma (MPM) had pfADA ≥35 IU/L compared to group medians of 10.6 IU/L and 16.8 IU/L respectively. The only causes of a pfADA over 100 were 7 cases of empyema which were all strongly neutrophil-predominant (≥80% minimum). None of the 44 transudative effusions (defined by Light’s criteria) had a pfADA ≥35 IU/L (median 5.7 IU/L). Notably, all 8 cases of lymphoma had pfADA <35 IU/L (median 20.4 IU/L, range 8.5–29.7). There was one case of mTB which fell into the low pfADA group (26.7 IU/L), a 41 year old male who was a current smoker.

**Table 3:** Causes of a high pfADA (≥35 IU/L).

Aetiology	N-	pfADA range (IU/L)	Lymphocyte predominant	Neutrophil predominant	Mean pleural pH (Range)
Mycobacterium tuberculosis	6	35.0–91.5	6 (100%)	0 (0%)	7.35 (7.10–7.40)
Complex parapneumonic effusion	13	35.7–98.1	1 (8%)	9 (69%)	6.96 (6.68–7.34)
Empyema	11	54.7–568.4	0 (0%)	10 (91%)	6.91 (6.67–7.24)
Malignant pleural mesothelioma	4	59.7–70.4	0 (0%)	1 (25%)	7.19 (7.17–7.33)
Non small cell lung cancer	2	36.8–85.4	0 (0%)	1 (50%)	7.20 (7.09–7.31)

### pfADA as a diagnostic test

Using a cut off of 35.0 IU/L, for all effusion types (n = 338), the sensitivity of pfADA for detecting mTB was 85.7% with a specificity of 90.9% and NPV of 99.7%. Area under the curve was 0.883 (95% CI; 0.732–1.000). Full results are shown in Table 4.

**Table 4:** Cross tabulation for the diagnostic performance of pfADA in all effusions (n = 338).

	Mycobacterium tuberculosis	No mycobacterium tuberculosis	
pfADA≥35 IU/L	6	30	Positive predictive value
			16.7%
			(95% CI; 6.4–32.8%)
pfADA<35 IU/L	1	301	Negative predictive value
			99.7%
			(95% CI; 98.2–99.9%)
	Sensitivity	Specificity	
	85.7%	90.9%	
	(95% CI; 42.2–97.6%)	(95% CI; 87.3–98.8%)	

When using the same cut-off in lymphocytic effusions alone (n = 98) NPV falls to 98.9%. Sensitivity remains the same and specificity improves to 98.9% with a greater area under the curve, 0.923 (95% CI; 0.770–1.000). Full results are shown in Table 5. See S3 Appendix for receiver operating curves (ROC) of both all effusion and lymphocytic effusions alone.

**Table 5:** Cross tabulation for the diagnostic performance of pfADA in lymphocytic effusions (n = 98).

	Mycobacterium tuberculosis	No mycobacterium tuberculosis	
pfADA≥35 IU/L	6	1	Positive predictive value
			85.7%
			(95% CI; 42.2–97.6%)
pfADA<35 IU/L	1	90	Negative predictive value
			98.9%
			(95% CI; 94.0–99.8%)
	Sensitivity	Specificity	
	85.7%	98.9	
	(95% CI; 42.2–97.6%)	(95% CI; 94.0–99.8%)	

## Discussion

The aim of this study was to test the ability of pfADA to aid in the diagnosis of tuberculous pleural effusions in an area where mTB incidence is low. In 2013 Public Health England (formerly the Health Protection Agency) reported the rate of mTB in South-West of England, where this study is based, as 7.8 per 100,000, in comparison to a national UK average of 14.6 per 100,000[8]. This is a similar incidence to other North-Western European counties e.g. France (8.2), Denmark (7.4), Germany (5.6)[12]. The main study finding was that a pfADA of less than 35 IU/L, regardless of effusion type, made a diagnosis of mTB very unlikely with a NPV of 99.7%. Additionally, patients with a lymphocytic effusion and a pfADA over 35 IU/L had a high likelihood of mTB (85% compared to a cohort prevalence of 2%).

### Previous literature

There have been several meta-analyses assessing the utility pfADA measurement, the largest carried out by Liang and colleagues who assessed 63 studies of varying size (28 to 600 patients). Despite including a large number of patients (n = 8093), their high values for sensitivity and specificity have little relevance to a low incidence population such as in the UK—the prevalence of mTB in our cohort was 2% (7/338) compared to an average prevalence of 34.5% in the meta-analysis. Differing methods of pfADA analysis (Giusti’s colorimetric method vs non-Giusti) are likely to have influenced the conclusions further still.

Porcel et al attempted to address the issue of varying methodologies by recruiting a large number of patients to a single trial in an intermediate incidence area (30 per 100,000)[11]. The authors used a Bayes’ formula technique to model a low prevalence situation. They concluded that as prevalence fell the positive predictive value would also fall, but negative predictive values would remain high. However, although this technique can allow for changes in mTB prevalence it makes assumptions as to the type of effusions that would then predominate. Another Spanish based study[13] used three time periods where incidence rates were different (thanks to a very effective mTB control programme) to examine the effect on pfADA’s diagnostic ability. They also concluded that the negative predictive value remains high, especially when used in combination with lymphocyte-predominance data. It should be noted that the lowest mTB rates in this study were still four times that of our cohort.

### Interpreting a low pfADA

In a low incidence population the likelihood of mTB is small, but patients with undiagnosed pleural effusions after routine investigations[14] and unable to undergo pleural biopsy may be given empirical anti-TB therapy if clinical suspicion remains high. This approach risks severe drug side effects in patients where the most likely alternative diagnosis is malignancy. In our study the likelihood of mTB fell to 0.3% in the context of a pfADA less than 35 IU/L in all cause effusions, and 1.0% in lymphocytic effusions. The strong performance of pfADA as a ‘rule out’ test means the use of empirical therapy is unlikely to be of benefit in this population group. As well as reducing patient harm, avoiding presumptive therapy is likely to be cost effective.

Previous literature would suggest low pfADA values should be viewed with caution in certain scenarios if clinical suspicion remains high. These include active smokers and increasing age[15]. Previous studies have attempted to stratify pfADA levels to patient age in tuberculous effusions[16, 17]. Unfortunately this study does not have the power to identify age specific reference ranges but also found that pfADA fell with age after multivariate analysis. Unlike some other diagnostic tests for mTB immunosuppression does not appear to affect pfADA levels[18].

### Interpreting a high pfADA

We also demonstrated that a pfADA over 35 IU/L detects mTB with good sensitivity and specificity. A patient with an effusion of malignant aetiology had only a 3% (6/172) chance of having a high pfADA and 0% (0/172) chance of the effusion also being lymphocyte predominant. The ability of pfADA to act as a ‘rule in’ test for mTB was greatly improved by consideration of the predominant cell type, increasing the specificity from 91% in all cause effusions to 99% in lymphocytic effusions.Six cases (2 NSCLC and 4 MPM) of malignant cause effusions had a high pfADA, although five appeared to have a co-existent pleural infection In other studies, high pfADA values have been reported in lymphoma[11, 19, 20], although all lymphoma cases in our cohort had pfADA values of less than 35 IU/L. Differentiating between mTB and lymphoma can be challenging, and additional tests such as lymphocyte subset analysis may help to distinguish one from the other[21]. A third of parapneumonic effusions had pfADA values of over 35 IU/L and in the case of empyema the result could be over 15 times the upper limit of normal. The phenomena of exceptionally high pfADA (e.g. >250 u/L) in empyema has been well documented[11, 13, 15].

### The place of pfADA in clinical practice in a low incidence population

Despite the good performance of pfADA in this study we would only support its use in certain clinical scenarios. Firstly, in patients for whom the cause of a pleural effusion is unclear after initial investigations but more invasive pleural biopsy is inappropriate the focus would be to use pfADA to exclude mTB and thereby avoid the need for empirical treatment. Secondly, in patients with lymphocytic effusions where mTB is strongly suspected but traditional investigations have proved inconclusive a high pfADA infers a strong likelihood of mTB and could prompt empirical anti-TB treatment. A high pfADA might raise the possibility of mTB where it previously seemed unlikely. However, a patient with a pfADA over 35 IU/L should still undergo a biopsy where possible to obtain microbiological confirmation and sensitivity data. This is especially important in areas of increasing resistance to first line antibiotics.

### Study limitations

Despite the large number of patients included in our study, due to low prevalence there were only 7 cases of mTB. Although the low numerator affects estimates of sensitivity, it has much less impact on the specificity and negative predictive value, and so assessments of the test’s ability to rule out mTB are likely to be accurate. Additionally, we only encountered cases of lymphocyte-predominant tuberculous effusion so are unable to comment on tuberculous effusions where neutrophils predominate. pfADA analysis was performed at an external laboratory, by an investigator blinded to patient diagnosis, and performed ‘en bloc’ post-recruitment. For this reason samples were stored frozen for a significant period of time and filtered before transit. Although pfADA has been shown to be highly stable[22] some studies have shown a small decline in the yield of pfADA after extended frozen storage[23]. However, storage time was not correlated with pfADA in our cohort. This study analysed the total pfADA from patient samples i.e. the sum of its isoforms ADA_1_ and ADA_2_. Some studies have supported the analysis of ADA_2_ alone as a marginally more specific analyte, as it reduces the false positive effect from neutrophilic effusions[24, 25]. However, the improved specificity of this isoform is superseded by consideration of the predominant cell type which we recommend for all baseline pleural fluid analysis. Not all clinical laboratories routinely report the differential cell counts of pleural fluid which affects the generalisability of this recommendation. We hope this study gives further weight to the value of cytology reports that routinely include differential cell counts.

### Summary

This is the first prospective study of the diagnostic accuracy of pfADA in a population of low mTB incidence. We have shown that a pfADA under 35 IU/L makes a diagnosis of mTB highly unlikely. A pfADA of over 35 IU/L in lymphocyte predominant effusions makes mTB the most likely diagnosis but does not replace pleural biopsy as the gold standard investigation. Based on these data we propose clinical scenarios where pfADA could be used in the work up of undiagnosed pleural effusions in areas of low mTB incidence. Further prospective studies to validate our findings would be beneficial.

## Supporting Information

S1 Appendix Diagnostic protocol for undiagnosed pleural effusions. (DOCX)

S2 Appendix Adenosine deaminase assay. (DOCX)

S3 Appendix Receiver operating curves. (DOCX)

## References

[1] Villegas MV, Labrada LA, Saravia NG (2000) Evaluation of polymerase chain reaction, adenosine deaminase, and interferon-gamma in pleural fluid for the differential diagnosis of pleural tuberculosis. Chest;118(5):1355–64. 11083686 10.1378/chest.118.5.1355

[2] Gopi A, Madhavan SM, Sharma SK, Sahn SA (2007) Diagnosis and treatment of tuberculous pleural effusion in 2006. Chest;131(3):880–9. 17356108 10.1378/chest.06-2063

[3] Valdes L, Alvarez D, San Jose E, Penela P, Valle JM, et al (1998). Tuberculous pleurisy: a study of 254 patients. Arch Intern Med;158(18):2017–21. 9778201 10.1001/archinte.158.18.2017

[4] Seibert AF, Haynes J Jr, Middleton R, Bass JB Jr (1991) Tuberculous pleural effusion. Twenty-year experience. Chest;99(4):883–6. 1901261 10.1378/chest.99.4.883

[5] Chakrabarti B, Ryland I, Sheard J, Warburton CJ, Earis JE (2006) The role of Abrams percutaneous pleural biopsy in the investigation of exudative pleural effusions. Chest;129(6):1549–55. 16778273 10.1378/chest.129.6.1549

[6] Diacon AH, Van de Wal BW, Wyser C, Smedema JP, Bezuidenhout J, et al (2003) Diagnostic tools in tuberculous pleurisy: a direct comparative study. Eur Respir J;22(4):589–91. 14582908 10.1183/09031936.03.00017103a

[7] McGrath EE, Anderson PB (2010) Diagnostic tests for tuberculous pleural effusion. Eur J Clin Microbiol Infect Dis;29(10):1187–93. 10.1007/s10096-010-0986-z 20556468

[8] Public Health England; Tuberculosis in the UK- 2013 report. Available: http://www.hpa.org.uk/webc/HPAwebFile/HPAweb_C/1317139689583. Accessed July 2014.

[9] Light RW, Macgregor MI, Luchsinger PC, Ball WC Jr (1972) Pleural effusions: the diagnostic separation of transudates and exudates. Ann Intern Med;77(4):507–13. 4642731 10.7326/0003-4819-77-4-507

[10] Maskell NA, Butland RJ, Pleural Diseases Group SoCCBTS (2003) BTS guidelines for the investigation of a unilateral pleural effusion in adults. Thorax;58 Suppl 2:ii8–17. 12728146 10.1136/thorax.58.suppl_2.ii8 PMC1766019

[11] Porcel JM, Esquerda A, Bielsa S (2010) Diagnostic performance of adenosine deaminase activity in pleural fluid: a single-center experience with over 2100 consecutive patients. Eur J Intern Med;21(5):419–23. 10.1016/j.ejim.2010.03.011 20816597

[12] World Health Organization (WHO) estimates of tuberculosis incidence by country (2012) (sorted by country). Available: http://wwwhpaorguk/webc/HPAwebFile/HPAweb_C/1317140584754. Accessed July 2014.

[13] Garcia-Zamalloa A, Taboada-Gomez J (2012) Diagnostic accuracy of adenosine deaminase and lymphocyte proportion in pleural fluid for tuberculous pleurisy in different prevalence scenarios. PLoS One;7(6):e38729 10.1371/journal.pone.0038729 22723878 PMC3377686

[14] Hooper C, Lee YC, Maskell N, Group BTSPG (2010) Investigation of a unilateral pleural effusion in adults: British Thoracic Society Pleural Disease Guideline. Thorax;65 Suppl 2:ii4–17. 20696692 10.1136/thx.2010.136978

[15] Lee SJ, Kim HS, Lee SH, Lee TW, Lee HR, et al (2014) Factors Influencing Pleural Adenosine Deaminase Level in Patients With Tuberculous Pleurisy. Am J Med Sci. 10.1097/MAJ.0b013e31828ffcd6 24762755

[16] Kapisyzi P, Argjiri D, Aliko A, Beli J, Vakeflliu Y, et al (2011) The Use of Different Cutoff Values of ADA Liquid Level in Diagnosis of Tuberculous Pleurisy in Countries With Different Incidence of Tuberculosis (Abstract) Chest;140(4_MeetingAbstracts):703A 10.1378/chest.1108296

[17] Tay TR, Tee A (2013) Factors affecting pleural fluid adenosine deaminase level and the implication on the diagnosis of tuberculous pleural effusion: a retrospective cohort study. BMC Infect Dis;13:546 10.1186/1471-2334-13-546 24238276 PMC3835552

[18] Chung JH, Kim YS, Kim SI, Park K, Park MS, et al (2004) The diagnostic value of the adenosine deaminase activity in the pleural fluid of renal transplant patients with tuberculous pleural effusion. Yonsei Med J;45(4):661–4. 15344207 10.3349/ymj.2004.45.4.661

[19] Yao CW, Wu BR, Huang KY, Chen HJ (2014) Adenosine deaminase activity in pleural effusions of lymphoma patients. QJM. 24854180 10.1093/qjmed/hcu106

[20] Antonangelo L, Vargas FS, Genofre EH, Oliveira CM, Teixeira LR, et al (2012) Differentiating between tuberculosis-related and lymphoma-related lymphocytic pleural effusions by measuring clinical and laboratory variables: is it possible? J Bras Pneumol;38(2):181–7. 22576425 10.1590/s1806-37132012000200006

[21] Bhatnagar R, Clive A, Zahan-Evans N, Morley A, Virgo P, et al (2013) The clinical utility of pleural lymphocyte subset analysis in undiagnosed effusions (abstract). Thorax;68(3):170–1.

[22] Antonangelo L, Vargas FS, Almeida LP, Acencio MM, Gomes FD, et al (2006) Influence of storage time and temperature on pleural fluid adenosine deaminase determination. Respirology;11(4):488–92. 16771922 10.1111/j.1440-1843.2006.00866.x

[23] Bielsa S, Esquerda A, Palma RM, Criado A, Porcel JM (2014) Influence of storage time on pleural fluid adenosine deaminase activity. Clin Lab;60(3):501–4. 24697129 10.7754/clin.lab.2013.130311

[24] Valdes L, San Jose E, Alvarez D, Valle JM (1996) Adenosine deaminase (ADA) isoenzyme analysis in pleural effusions: diagnostic role, and relevance to the origin of increased ADA in tuberculous pleurisy. Eur Respir J;9(4):747–51. 8726940 10.1183/09031936.96.09040747

[25] Zemlin AE, Burgess LJ, Carstens ME (2009) The diagnostic utility of adenosine deaminase isoenzymes in tuberculous pleural effusions. Int J Tuberc Lung Dis;13(2):214–20. 19146750

