# Supplementary material for: Pleural Fluid Adenosine Deaminase (Pfada) in the Diagnosis of Tuberculous Effusions in a Low Incidence Population
Source: PLoS One. 2015 Feb 3;10(2):e0113047. doi: 10.1371/journal.pone.0113047 (PMC4315514; doi:10.1371/journal.pone.0113047)
Supplement: S1 Appendix — (DOCX) [file pone.0113047.s001.docx]

**Appendix S1: Diagnostic protocol for undiagnosed pleural effusions.**

**Malignant**

- Malignant pleural fluid cytology or biopsy, or
- Histologically confirmed pleural malignancy or extra- thoracic/pulmonary malignancy with radiographic evidence of metastasis to ipsilateral pleura on CT, or
- Radiological changes meeting Leung’s criteria[1] which have progressed in keeping with malignancy on interval CT scan in the correct clinical context, or
- Autopsy confirming pleural malignancy

**Simple Parapneumonic effusion**

Clinical presentation suggestive of sepsis with appropriate chest radiology and pleural fluid which is gram stain and culture negative with a pH >7.2 and an absence of loculation on thoracic ultrasound, and

Resolution of effusion on CXR after antibiotics or clinical progression to pleural infection (see below)

**Complicated Parapneumonic Effusion**

Clinical presentation suggestive of sepsis, and

- Pleural fluid pH ≤7.2 or pleural fluid loculation on ultrasound and follow up for at least 6months inconsistent with pleural malignancy, or
- Pleural fluid gram stain or culture positive, or
- Pleural infection confirmed by pleural biopsy histology and/or microbiological culture, or
- CT scan consistent with pleural infection with radiological resolution following treatment with antibiotics.

**Empyema**

Features of a complicated parapneumonic effusion with frank pus seen at thoracoscopy or thoracocentesis.

**Connective tissue disease (including RA)
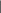
**

Systemic features or known diagnosis of connective tissue disease, and

Chest radiology (including CT imaging) showing benign features (e.g. doesn’t meet any of Leung’s criteria) with at least 6 months follow-up and /or pleural biopsy negative for malignancy.

**Pulmonary embolism**

Evidence of PE on CTPA, and

No alternative explanation for pleural effusion on cross sectional imaging or pleural fluid analysis. (NB the CT shows no evidence of pleural thickening – which would suggest another cause)

**BAPE or diffuse pleural thickening due to asbestos**

History of asbestos exposure or evidence of pleural plaques on CT, and

- Stable or improving CT appearances with follow-up for at least 12 months (the development of enfolded lung is allowed), or
- Negative thoracoscopy (benign pleural biopsy)

**Congestive Cardiac Failure**

- History and examination features of CCF, or
- Evidence of at least moderate LV systolic or diastolic failure or severe valvular disease on echo, or
- Improvement of effusion and symptoms with diuretic therapy

**Coronary artery bypass graft (CABG)**

CABG in 3 months prior to development of pleural effusion in the absence of an alternative cause

**Hepatic hydrothorax**

Known history or clinical presentation consistent with liver disease, and

Recurrent transudative pleural effusion with negative cytology.

**Renal failure or hypoalbuminaemia**

Biochemical confirmation of renal failure or hypoalbuminaemia in the absence of clinical, radiological or pleural fluid analysis suspicious of an alternative cause.

**TB pleuritis**

Resolution of presenting pleural effusion after 6months anti-TB therapy, AND

- Positive fluid or tissue culture for mycobacterium tuberculosis (mTB), or
- Acid fast bacilli confirmed in sputum, pleural fluid or pleural tissues, or
- Patients with a strong clinical and radiological suspicion of TB pleuritis and a positive quantiferon.

**Inflammatory pleuritis (Non-specific pleuritis)**

Demonstration of non-specific inflammatory pleuritis on pleural biopsy, and

Follow-up for 12 months without progression that would suggest a malignant cause.

**Idiopathic**

- Exhaustive investigations including 12 months follow-up with interval CT scans has not demonstrated a diagnosis, or
- Patient unfit for further investigation and follow up, or
- Patient died without definitive diagnosis and no post mortem examination conducted

**Other**

Pathologies falling outside this protocol were diagnosed based on well-established clinical criteria and guidelines.

1. Leung AN, Muller NL, Miller RR. CT in differential diagnosis of diffuse pleural disease. AJR Am J Roentgenol. 1990 Mar;154(3):487-92. PubMed PMID: 2106209.
