# Supplementary material for: Pleural Fluid Adenosine Deaminase (Pfada) in the Diagnosis of Tuberculous Effusions in a Low Incidence Population
Source: PLoS One. 2015 Feb 3;10(2):e0113047. doi: 10.1371/journal.pone.0113047 (PMC4315514; doi:10.1371/journal.pone.0113047)
Supplement: S2 Appendix — (DOCX) [file pone.0113047.s002.docx]

**Appendix S2: Adenosine deaminase assay.**

The adenosine deaminase assay was performed by a single investigator (with over 30 years experience) blinded to patient diagnosis, in a lab geographically separate to site of recruitment.

In summary, 25 μl of fluid was incubated in a 1.5 ml Eppendorf tube at 37^o^c, in 50 mmol/L sodium phosphate buffer pH 7.4 and 3.75 mmol/L adenosine (total volume 200 μl). Assays were performed in duplicate. After 30 minutes the reaction was stopped by adding 25 μl 40% w/v trichloroacetic acid, and spun for 2min in a microfuge. The supernatant was transferred to a clean tube and the trichloroacetic acid was back-extracted with water-saturated diethylether..

Ten microlitres of the supernatant was injected onto a Waters Alliance HT -2795 system with a Photodiode Array UV detector, with a 150x4.6 mm ODS (C18)- 5u column, and eluted isocratically with an ion-pair buffer (5 mmol/L tetrabutylammonium chloride, 40 mmol/L ammonium acetate, pH 2.75) at 1 ml/min. ,run time 6 min.. The activity was calculated from the two product peaks, inosine and hypoxanthine, and a blank (zero time), subtracted.
