# Supplementary material for: Pleural Fluid Adenosine Deaminase (Pfada) in the Diagnosis of Tuberculous Effusions in a Low Incidence Population
Source: PLoS One. 2015 Feb 3;10(2):e0113047. doi: 10.1371/journal.pone.0113047 (PMC4315514; doi:10.1371/journal.pone.0113047)
Supplement: S3 Appendix — (DOCX) [file pone.0113047.s003.docx]

**APPENDIX S3; RECEIVER OPERATING CURVES.**


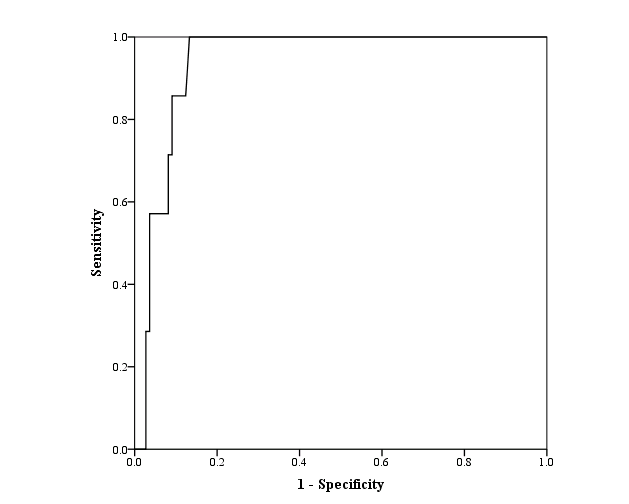
A; Diagnostic value of pfADA for tuberculous effusions for all cause effusions (n=338) using receiver operating characteristic (ROC) curve analysis. AUC= 0.883.


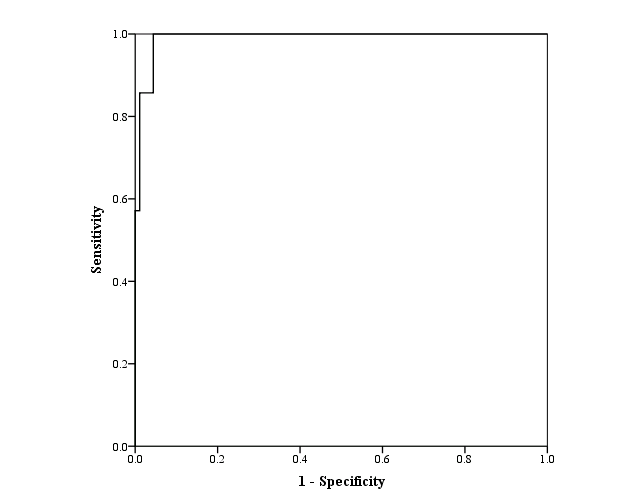
B; Diagnostic value of pfADA for tuberculous effusions for lymphocytic effusions (n=98) using receiver operating characteristic (ROC) curve analysis. AUC = 0.923.
